# Urine Neutrophil Gelatinase-Associated Lipocalin a Possible Diagnostic Marker for Egyptian Hepatocellular Carcinoma Patients

**DOI:** 10.31557/APJCP.2020.21.8.2259

**Published:** 2020-08

**Authors:** Eman Abdelsameea, Ali Nada, Nabil Omar, Saleh M Saleh, Mary Naguib, Hosam El-Din M El-Ezawy, Lamiaa Bakry, Maha Elsabaawy

**Affiliations:** 1 *Hepatology and Gastroenterology, National Liver Institute, Menoufia University, Egypt. *; 2 *Department of Clinical Pathology, National Liver Institute, Menoufia University, Egypt. *; 3 *Department of Clinical Biochemistry and Molecular Diagnostics, National Liver Institute, Menoufia University, Egypt. *

**Keywords:** Hepatocellular carcinoma (HCC), urinary neutrophil gelatinase, associated lipocalin (NGAL), Liver cirrhosis

## Abstract

**Background:**

Most effective method for reducing mortality from hepatocellular carcinoma (HCC) is early diagnosis. Despite its lack of adequate sensitivity, ultrasound is considered fundamental for HCC screening.

**Aim:**

to evaluate urinary neutrophil gelatinase-associated lipocalin (NGAL) as non-invasive marker for HCC diagnosis in Egyptian patients.

**Methods:**

One hundred and twenty patients were divided into three groups (40 patients each): patients with chronic viral hepatitis (HCV or HBV), cirrhotic patients and HCC patients and 40 healthy age and gender matched subjects were enrolled as control group. After clinical assessments, urinary NGAL was measured by enzyme-linked immunosorbent assay.

**Results:**

Our results revealed that median level of urinary NGAL was 290, 834, 1090 and 1925 pg/ml in control, chronic hepatitis, cirrhotic and HCC groups respectively among studied groups (p<0.001). Receiver operating characteristics (ROC) analysis showed that urinary NGAL cutoff value of 1255 ng/ml could discriminate between HCC and cirrhosis. The area under curve (AUC) was 0.95 with 90% sensitivity, 87.5% specificity (p-value <0.001). In HCC group, urine NGAL level didn`t show significant correlation with Child Pugh score, MELD score or Barcelona Clinic Liver Cancer (BCLC) stage.

**Conclusion:**

Urinary NGAL could be a simple, non-invasive test for diagnosis of HCC in chronic liver disease patients.

## Introduction

Lipocalin 2 (LCN2), a member of the lipocalin subfamily, also known as neutrophil gelatinase-associated lipocalin (NGAL), is a 25 kDa secreted glycoprotein (Bauvois and Susin, 2018). NGAL also has other forms in serum; 30 kDa isoform which probably results from differential glycosylation, 46 kDa disulfide-linked homodimer and 130 kDa heterodimer which is bound to the inactive form of the matrix metalloproteinase-9 (proMMP-9) (Bouchet and Bauvois, 2014).

MMP9 plays an important role in tumor invasion and metastasis. Studies have shown that (NGAL/MMP9) bound form protects MMP9 from degradation thus increases its activity (Yan et al., 2001; Liu et al., 2015). 

Actually, NGAL was first discovered inside the specific granules of neutrophils as a part of matrix metalloproteinase-9 (MMP-9) (Yang and Moses, 2009). However later studies have shown that lipocalin 2 is synthesized in other tissues as; the kidney under conditions of tubular injury and the liver during liver cell injury or regeneration (Borkham-Kamphorst et al., 2013). Thus NGAL may be a potential biomarker of liver injury.

Hepatocellular carcinoma (HCC) is one of the most common ten solid cancers worldwide and is considered the second cause of death from malignancy (Mazzanti et al., 2016) with incidence still increasing in many countries. The most effective way of reducing mortality due to HCC is prevention (Ferlay et al., 2015).

As an inflammation-driven disease, patients with chronic infections as chronic hepatitis B virus (HBV) or hepatitis C virus (HCV) infections are at greater risk of developing HCC. Thus they should be continuously monitored (Ziada et al., 2016). Ultrasound examination and alpha-fetoprotein are still fundamental tests used for surveillance for HCC despite their lack of adequate sensitivity (Attwa and El-Etreby, 2015). 

Since some markers not originating from the kidney may appear in the urine through glomerular filtration (Ariza et al., 2015). Thus this study aims to assess urinary lipocalin as a promising candidate for diagnosis of HCC in chronic liver disease patients 

## Materials and Methods

*Subjects and Methods*

*Study participants*

The present case control study was conducted on 120 patients recruited from the outpatient clinic and inpatient unit of hepatology and gastroenterology department, National Liver Institute, Menoufia University. Patients were divided into three groups (40 patients each): chronic viral hepatitis (HCV or HBV) patients, cirrhotic patients and HCC patients. Forty healthy subjects were enrolled as control group. 

Diagnosis of HCC was confirmed by multislice triphasic computed tomography (CT) scan with or without elevated alpha fetoprotein more than 200 ng/ml. Patients older than 70 years or younger than 18 years, patients with renal impairment (serum creatinine > 1.5 mg /dL), patients presenting with history of gastrointestinal bleeding within 7 days before inclusion in the study and patients with any kind of infection or liver tumors (e. g. adenoma) were excluded from the study.

Study protocol was approved by the local ethical committee of National Liver Institute and an informed consent was obtained from all subjects.

*Routine laboratory assessment*

After full history taking with clinical examination, routine laboratory tests were done. Liver tests including alanine aminotransferase (ALT), aspartate aminotransferase (AST), alkaline phosphatase (ALP), Gamma glutamyl transferase (GGT), total bilirubin, albumin and total protein and renal tests including serum urea and creatinine were done on Cobas Integra 400 auto analyzer, Hoffman La Roche Company, Switzerland. Prothrombin concentration and international normalized ratio (INR) were analyzed via Thromboreal S, Behring fibrin timer II, Behring Inc., 1999, Germany. Serum alpha fetoprotein (AFP) was detected by Cobas e411 immunoassay analyzer (Roche diagnostics- Gm bH, D- 68305Mannhein, Germany).

*Enzyme-linked Immunosorbent Assay (ELISA)*

Determination of urinary NGAL level was performed using solid phase enzyme-linked immunosorbent assay (ELISA) provided by NGAL, Bio PORTO, ELISA Kit (Bio PORTO Diagnostics A/S Denmark). Mid- stream urine samples (10 ml for each sample) were collected in a clean dry container. They were centrifuged, examined microscopically to exclude urinary tract infection. The supernatant from the sample was stored at -20 until used in the ELISA analysis according to the manufacturer’s recommended procedure.

*Statistical analysis*

Results were statistically analyzed by using statistical package of social sciences (SPSS 22.0, IBM/SPSS Inc., Chicago IL). Mean and standard deviation (SD) was used for summarizing continuous data while median and interquartile range (IQR) for skewed data. Categorical data was expressed as frequency with percentage. For continuous variables, Kruskal-Wallis test (a non-parametric equivalent for ANOVA) was used to compare between several groups when normality and homogeneity assumptions were violated. For multiple pairwise comparisons, Bonferroni Post Hoc test was used with significant Kruskal-Wallis test. The Chi-square (x^2^) test was used to compare categorical variables. Spearman correlation coefficient (rs) was calculated to indicate the strength of association between not normally distributed numerical variables. Receiver operating characteristic (ROC) analysis was used to assess diagnostic performance of urinary NGAL for HCC detection. 

**Table 1:** Demographic Data and Laboratory Investigations of All Studied Groups

Parameters	Control	Chronic hepatitis	Cirrhosis	HCC	Kruskal-Wallis test
	(n = 40)	(n=40 )	(n = 40)	(n = 40)	*P*-value
Age (years)	b, c, d	a, d	a, d	a, b, c	<0.001
Median (IQR)	46.0 (4.0)	35.0 (19.0)	43.0 (10.0)	57.5 (11.0)	
min-max	40.0 - 54.0	19.0 - 56.0	23.0 - 56.0	42.0 - 77.0	
Gender [n (%)]					0.116
Male	20 (54.1)	27 (67.5)	21 (52.5)	30 (75.0)	
Female	17 (49.5)	13 (32.5)	19 (47.5)	10 (25)	
AST (U/L)	b, c, d	a, c	a, b, d	a, c	<0.001
Median (IQR)	14.0 (3.0)	41.0 (10.0)	89.0 (20.0)	45.0 (26.5)	
min-max	10.0 - 19.0	28.0 - 80.0	56.0 - 165.0	22.0 - 89.0	
ALT (U/L)	b, c, d	a, d	a, d	a, b, c	<0.001
Median (IQR)	15.0 (3.0)	54.5 (31.8)	60.5 (14.8)	40.0 (22.5)	
min-max	11.0 - 20.0	30.0-120.0	41.0 - 85.0	20.0 - 75.0	
ALP (U/L)	c	c, d	a, b, d	b, c	<0.001
Median (IQR)	46.0 (5.5)	22.0 (17.3)	109.0 (37.0)	75.0 (75.0)	
min-max	42.0 - 56.0	12.0 - 260.0	56.0 - 167.0	24.0 - 270.0	
GGT (U/L)	c, d	c, d	a, b	a, b	<0.001
Median (IQR)	27.0 (10.0)	22.0 (11.0)	42.0 (11.8)	55.0 (29.8)	
min-max	11.0 - 46.0	9.0 - 47.0	21.0 - 50.0	16.0 - 228.0	
Total bilirubin (mg/dL)	b, c, d	a, c, d	a, b, d	a, b, c	<0.001
Median (IQR)	1.0 (0.2)	0.5 (0.4)	3.4 (1.9)	2.0 (0.8)	
min-max	0.8 - 1.1	0.1 - 1.1	1.0 - 6.8	0.7 - 8.0	
Albumin (g/dL)	c, d	c, d	a, b	a, b	<0.001
Median (IQR)	4.2 (0.4)	4.1 (0.4)	2.1 (0.3)	2.6 (0.3)	
min-max	3.8 - 4.9	3.5 - 4.9	1.8 - 2.9	1.5 - 3.1	
TP (g/dL)	b, c, d	a	a	a	<0.001
Median (IQR)	7.5 (0.8)	7.0 (0.6)	6.8 (0.7)	7.0 (1.7)	
min-max	6.7 - 8.4	6.0 - 8.0	5.6 - 8.0	4.5 - 8.0	
Urea (mg/dL)	c	c, d	a, b, d	b, c	<0.001
Median (IQR)	32.0 (9.5)	25.0 (2.8)	55.0 (14.8)	35.0 (21.5)	
min-max	17.0 - 42.0	22.0 - 35.0	40.0 - 90.0	13.0 - 145.0	
Creatinine (mg/dL)	b	a, c, d	b, d	b, c	<0.001
Median (IQR)	0.9 (0.2)	0.6 (0.4)	1.0 (0.3)	0.8 (0.3)	
min-max	0.6 - 1.1	0.2 - 1.2	0.6 - 1.4	0.5 - 3.0	
INR	c, d	c, d	a, b	a, b	<0.001
Median (IQR)	1.0 (0.1)	1.0 (0.1)	1.4 (0.2)	1.3 (0.3)	
min-max	0.9 - 1.3	0.9 - 1.3	1.1 - 2.3	1.1 - 2.0	

IQR, Interquartile range; ALT, Alanine aminotransferase; AST, Aspartate aminotransferase; ALP, alkaline aminotransferase; GGT, Gamma glutamyl transferase; TP, Total Protein; INR, International normalized ratio; * χ^2^, Pearson chi square test; Superscripts a, b , c , d denotes that parameter distribution in the assigned group differs significantly from control, HCV, Cirrhosis, and HCC, respectively at < 0.05 level; p-values for multiple pairwise comparisons adjusted by Bonferroni Post hoc test

**Table 2:** Median Levels of Urine NGAL and Serum AFP among the Different Studied Groups

Parameters	Control	Chronic hepatitis	Cirrhosis	HCC	*P*-value
	(n = 40)	(n=40 )	(n = 40)	(n = 40)	
NGAL (pg/mL)	b, c, d	a, d	a, d	a, b, c	<0.001
Median (IQR)	290.0 (240.0)	834.0 (552.5)	1090.0 (227.5)	1825.0 (4512.5)	
min-max	26.0 - 590.0	338.0 - 2120.0	260.0 - 1500.0	1130.0 - 9960.0	
AFP (ng/mL)	b, c, d	a, c, d	a, b, d	a, b, c	<0.001
Median (IQR)	0.9 (0.3)	1.4 (0.9)	14.0 (15.5)	234.0 (438.2)	
min-max	0.6 - 2.0	0.7 - 3.0	2.3 - 35.0	2.4 - 789.0	

NGAL, neutrophil gelatinase-associated lipocalin; AFP, Alpha fetoprotein; IQR, Interquartile range; Superscripts a, b , c , d denotes that parameter distribution in the assigned group differs significantly from control, HCV, Cirrhosis, and HCC, respectively at the 0.05 level ;p-values for multiple pairwise comparisons adjusted by Bonferroni Post hoc test

**Table 3:** Diagnostic Performance of Urine NGAL and Serum AFP for Discrimination between HCC and Cirrhosis Groups

	AUC	Sensitivity (%)	Specificity (%)	PPV (%)	NPV	SE	Accuracy %	*P*-value
Urine NGAL Cutoff (1,255 ≥ pg/mL)	0.95	90	87.5	88.6	89	0.02	88.8	< 0.001
AFP Cutoff (≥ 39.6 ng/mL)	0.92	85	100	100	86.1	0.04	92.5	< 0.001

HCC, Hepatocellular carcinoma; AFP, Alpha fetoprotein; NGAL, neutrophil gelatinase-associated lipocalin; AUC, Area under curve; SE, Standard error; PPV, Positive predictive value; NPV, Negative predictive value

**Figure 1:**
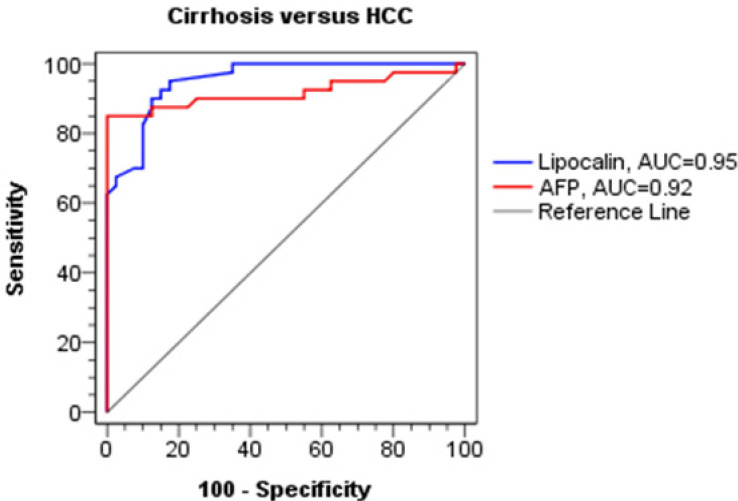
ROC Curves of Serum AFP and Urine NGAL for Dicrimination between Cirrhosis and HCC Groups

**Table 4:** Combined Urine NGAL and Serum AFP for Discrimination between HCC and Cirrhosis Groups

Test characteristics	Combined measurement of AFP and urine NGAL Cirrhosis vs. HCC
Best cutoff value (probability)	≥ 0.725
AUC	0.997
SE	0.003
p-value	< 0.001
Sensitivity %	95
Specificity %	100
PPV %	100
NPV %	94.87
Accuracy %	97.5

HCC, Hepatocellular carcinoma; AFP, Alpha fetoprotein; NGAL, neutrophil gelatinase-associated lipocalin; AUC, Area under curve; SE, Standard error; PPV, Positive predictive value; NPV, Negative predictive value

**Table 5:** Relation between Urine NGAL and Serum AFP with Number of Focal Lesions in HCC Groups

Parameters	Single	Multiple	Mann-Whitney U test	*P*-value
	(n = 23)	(n=17 )	Z	
Urine NGAL (pg/mL)			0.89	0.374 NS
Median (IQR)	1800.0 (2610.0)	3200.0 (5995.0)		
min-max	1130.0 - 9960.0	1200.0 - 9560.0		
AFP (ng/mL)			0.38	0.702 NS
Median (IQR)	247.0 (454.0)	221.0 (455.9)		
min-max	2.4- 789.0	9.3 - 595.0		

IQR, Interquartile range; NS, Non-significant at p-value ≥ 0.05; AFP, Alpha fetoprotein; NGAL, neutrophil gelatinase-associated lipocalin; HCC, Hepatocellular carcinoma

**Figure 2:**
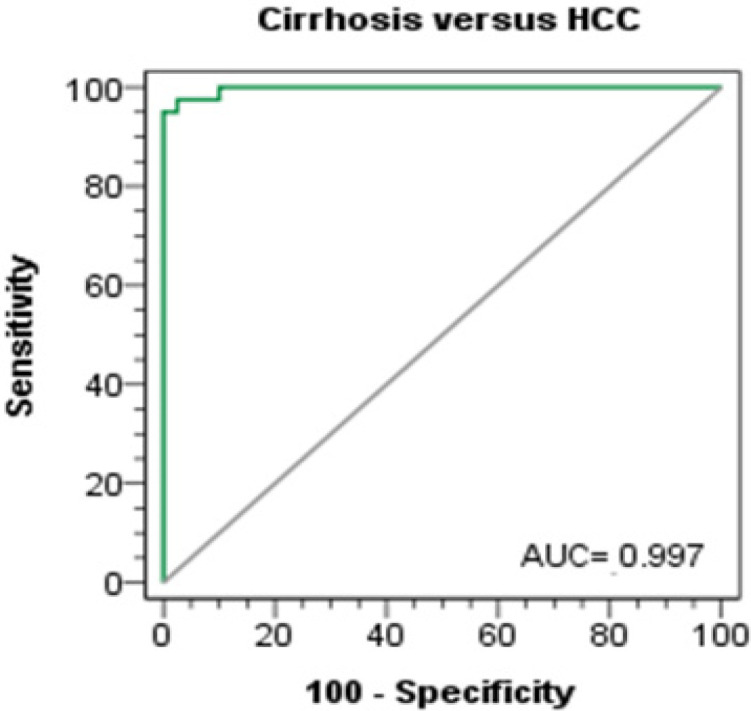
ROC Curves of Combined Serum AFP and Urine NGALfor Dicrimination between Cirrhosis and HCC Groups

**Table 6:** Correlation between Urine NGAL and Child Score, MELD, BCLC and Foci Size in HCC Group

Parameters correlated	Urine NGAL	
	HCC (n=40)	
	*r*_s_	*P*-value
Child score	0.17	0.283 NS
MELD score	-0.22	0.174 NS
BCLC stage	-0.15	0.362 NS
Foci size	-0.06	0.703 NS

*r*_s_ *, *Spearman correlation coefficient; NS, Non significant at p-value ≥ 0.05; HCC, Hepatocellular carcinoma; NGAL, neutrophil gelatinase-associated lipocalin; MELD, Model for end stage liver disease; BCLC, Barcelona Clinic Liver Cancer

## Results

*Demographic data and laboratory investigations of all studied groups*

Clinical characteristics of the studied groups were shown in Tables 1. Median ages were 46, 35, 43 and 57 years old in control, chronic hepatitis, cirrhotic and HCC groups respectively showing significant difference among them. However, there was no significant difference regarding gender distribution. 

 On the other hand, there was significant difference among different groups regarding liver tests (AST, ALT, ALP, GGT, total bilirubin, albumin, total protein and INR) and renal tests (urea and creatinine) with p value <0.001 (Table 1). 

*Urine NGAL and serum alpha fetoprotein levels among studied groups*

In control group, urine NGAL level ranged from 26- 590 pg/ml with median level 290 pg/ml, while in chronic hepatitis group, the value ranged from 338- 2120 pg/ml with median level 834 pg/ml. Values in cirrhotic patients group ranged from 260- 1500 pg/ml with median value 1090 pg/ml and in HCC group the range was 1,130-9960 pg/ml with median level 1825 pg/ml. 

Level of urine NGAL showed significant difference (p-value <0.001) among all studied groups mutually, except on comparing between chronic hepatitis and cirrhotic patients groups there was no significant difference between both groups.

On the other hand, studying serum levels of AFP revealed that there was significant difference among the four studied groups (p-value <0.001). In control group, serum AFP ranged from 0.6- 2 ng/ml with median value 0.9 ng/ml, in chronic hepatitis group values ranged from 0.7- 3.0 ng/ml with median 1.4 ng/ml, in cirrhotic patients group it ranged from 2.3- 35 ng/ml with median value 14 ng/ml and in HCC group AFP level ranged from 2.4- 789 ng/ml with median 234 ng/ml (Table 2).

*Studying diagnostic performance of urinary NGAL and serum AFP for discrimination between HCC and cirrhosis groups*

Receiver operating characteristics (ROC) analysis showed that urinary NGAL cutoff value of 1255 ng/ml could discriminate between HCC and cirrhosis. The area under curve (AUC) was 0.95 with 90% sensitivity, 87.5% specificity (p-value <0.001) Table 3 and Figure 1. 

Binary logistic regression analysis was done to evaluate combined measurement of urinary NGAL and serum AFP levels. It showed that AUC was 0.997 with 95% sensitivity and 100% specificity (p-value <0.001) Table 4 and Figure 2.

However, there was no correlation between urine NGAL level and the number of focal lesions in HCC group (p= 0.374) as shown in Table 5. Also, there was no correlation between serum AFP and the number of focal lesions in HCC group (p= 0.702) as shown in Table 5. In HCC group, urine NGAL level didn`t show correlation with Child Pugh score, MELD score or Barcelona Clinic Liver Cancer (BCLC) stage (Tables 6). 

The number of patients diagnosed as HCC with AFP < 200 ng/ml when using urine NGAL at level ≥ 1255 pg/mL was 15 while the number of patients diagnosed as HCC with AFP ≥ 200 ng/ml when using urine NGAL at level ≥ 1255 pg/mL was 21 showing no significant difference at that cutoff value for AFP (p= 0.638).

## Discussion

Lipocalin 2 (NGAL) is a member of diverse protein family. They share highly conserved lipocalin folds that are composed of eight- stranded, antiparallel β-barrel which forms an internal binding site. This allows lipocalins to bind and transport various hydrophobic ligands. In addition, they are secreted from different tissues, thus have role in various functions (Asimakopoulou and Weiskirchen, 2015).

NGAL forms complex with matrix metalloproteinase 9 (MMP-9) increasing its stability. MMP-9 was stated to have role in degradation of basement membrane and extracellular matrix. Thus, MMP-9/NGAL complex may contribute to tumor progression and metastasis (Hu et al., 2018).

In addition, NGAL has role in iron-depletion bacteriostatic strategy. Enterochelin is released by bacteria into the extracellular environment to capture iron. NGAL can competitively bind to the secreted enterochelin and the iron-bound form of NGAL is internalized in the host cell. Thus it has a role in innate immunity (Goetz et al., 2002; Singer et al., 2013). However, this leads to subsequent increase of intracellular iron. Iron, in turn, is essential for controlling cellular proliferation, invasion and cancer metastasis (Jung et al., 2017).

Thus, our study aimed to assess urine NGAL as a promising candidate for diagnosis of HCC in chronic liver disease patients. 120 patients participated in the study; they were subgrouped to chronic viral hepatitis (HCV or HBV) patients, cirrhotic patients and HCC patients. Forty healthy subjects were enrolled as control group.

Urinary NGAL was significantly elevated in HCC group compared to cirrhotic patients group, chronic hepatitis group and control group (p value <0.001). This was in accordance with the study directed by Abd El Moety et al., (2013). They found that lipocalin 2 in the peripheral blood of chronic HCV Egyptian patients with HCC was significantly elevated compared to chronic HCV group and control (p-value <0.001). 

Further they noticed that there was positive correlation between lipocalin 2 (LCN2) and MMP-9 and negative correlation with tissue inhibitors of MMP (TIMPs) in the 3 studied groups suggesting that this may have role in progression of cirrhosis and hepatocellular carcinoma (Abd El Moety et al., 2013).

To our knowledge, this is the first study evaluating NGAL in urine as a non- invasive diagnostic marker for HCC. Previously Ariza and colleagues stated that LCN2, a small protein expressed in several tissues due to injury, could be filtered in glomeruli and assessed in urine. Accordingly they studied LCN2 in plasma and urine as diagnostic marker of acute on top chronic liver failure (ACLF) in cirrhotic patients. They reported that plasma and urine LCN2 were significantly elevated in ACLF group vs. non ACLF group. Urine LCN2 was an independent predictive factor of ACLF and of 28 day transplant-free mortality (Ariza et al., 2016).

Our study revealed that NGAL in urine was significantly elevated in cirrhotic patients group compared to control group. Also, Kim et al reported that urine NGAL correlates with degree of fibrosis according to METAVIR score suggesting that this reflect the activity of urine MMP-9 (Kim et al., 2010).

On the other hand, in HCC group, urine NGAL levels didn’t show correlation with Child Pugh score, MELD score or BCLC stage. Also, it showed no correlation with number of focal lesions.

We found that urine NGAL cut off value of 1255 pg/ml could distinguish patients with HCC from cirrhotic patients with sensitivity 90% and specificity 87.5% (p-value <0.001) and when it was combined with serum AFP level, they had a better sensitivity 95% and specificity 100%. Thus urine NGAL could be a possible noninvasive marker added to the current measures used for continuous monitoring of chronic hepatitis patients for diagnosis of HCC. 

*Ethics approval and consent to participate*

The study was conformed to the ethical guidelines of the 1975 Declaration of Helsinki and was approved by the institutional review board of National Liver Institute, Menoufia University, Egypt. Written informed consents were obtained from all studied subjects. 

## Data Availability

Data used to support the findings of this study are included within the article.
